# Methods Used for the Eradication of Staphylococcal Biofilms

**DOI:** 10.3390/antibiotics8040174

**Published:** 2019-10-04

**Authors:** Maciej Jaśkiewicz, Adriana Janczura, Joanna Nowicka, Wojciech Kamysz

**Affiliations:** 1 Department of Inorganic Chemistry, Faculty of Pharmacy, Medical University of Gdańsk, 80-416 Gdańsk, Poland; kamysz@gumed.edu.pl; 2 Department of Microbiology, Faculty of Medicine, Medical University, 50-368 Wrocław, Poland; adriana.janczura@umed.wroc.pl (A.J.); joanna.nowicka@umed.wroc.pl (J.N.)

**Keywords:** *Staphylococcus aureus*, staphylococci, biofilm, antimicrobial susceptibility, biofilm eradication

## Abstract

*Staphylococcus aureus* is considered one of the leading pathogens responsible for community and healthcare-associated infections. Among them, infections caused by methicillin-resistant strains (MRSA) are connected with ineffective or prolonged treatment. The therapy of staphylococcal infections faces many difficulties, not only because of the bacteria’s resistance to antibiotics and the multiplicity of virulence factors it produces, but also due to its ability to form a biofilm. The present review focuses on several approaches used for the assessment of staphylococcal biofilm eradication. The methods described here are successfully applied in research on the prevention of biofilm-associated infections, as well as in their management. They include not only the evaluation of the antimicrobial activity of novel compounds, but also the methods for biomaterial functionalization. Moreover, the advantages and limitations of different dyes and techniques used for biofilm characterization are discussed. Therefore, this review may be helpful for those scientists who work on the development of new antistaphylococcal compounds.

## 1. Introduction

*Staphylococcus aureus* is one of the leading pathogens associated with nosocomial infections and the development of new antibiotics to eradicate it is urgently needed [1]. First of all, these bacteria can develop resistance to almost all antibiotics that have ever been used. Second, *S. aureus* can be an asymptomatic colonizer of healthy individuals [2]. For instance, approximately 30% of humans (with a slight dominance of healthcare workers) are nasal carriers of these bacteria as part of their normal flora [3,4]. These individuals are considered to be the major source of *S. aureus* prevalence in the environment. However, transmission may take place not only as a result of direct contact with a colonized or infected individual, but also through contaminated objects, such as doors, public transport handles, bed linen, or even blood pressure cuffs [5,6].

The *S. aureus* carriers will not get infected if basic hygiene principles are maintained, but several factors, such as the loss of a skin barrier, diabetes, or immune system disorders, may predispose one to the infection. Unlike other *Staphylococcus* species, *S. aureus* was found to be pathogenic in the absence of obvious predisposing host conditions, such as general immune system suppression or local immunodeficiency associated with the presence of a foreign material [7]. It should also be noted that colonization does not always result in a disease but *S. aureus* carriers are certainly prone to staphylococcal infections [8].

The diseases caused by *S. aureus* can be divided into two categories: community and hospital-acquired. Therefore, the same division is also applied for methicillin-resistant strains (MRSA) when the etiology of a particular infection is being determined. For this purpose, two terms have been adopted: healthcare-associated MRSA (HA-MRSA) and community-associated MRSA (CA-MRSA) [9]. Skin and soft-tissue infections (SSTIs) are the most frequent ones associated with the presence of *S. aureus*. Moreover, recent reports highlight an increase in multi-resistant organisms (especially MRSA) in both community- and hospital-acquired SSTIs [10,11,12,13,14]. Skin infections can also be associated with other dermatological disorders, such as atopic dermatitis (AD) [15,16]. Nevertheless, if only the basic treatment becomes ineffective, these skin diseases can progress to bacteremia, bloodstream infections, endocarditis, or even sepsis [17].

Apart from SSTIs, *S. aureus* is considered to be the most common pathogen in osteoarticular infections, such as osteomyelitis, prosthetic joint infections, and native joint arthritis [18,19,20,21,22,23]. Moreover, it should be noted that staphylococci can invade osteoblasts and can survive in a metabolically inactive state without affecting the host cells. There, they can persist in small colony variants (SCVs) that may subsequently lead to recurrent infections [24,25].

Infective endocarditis (IE) is another disease where *S. aureus* plays a crucial role. As a matter of fact, *S. aureus* is a common etiological factor of IE and is associated with nearly 30% of all its cases [18,26,27]. The majority of these infections involve staphylococcal growth on aortic or mitral valves and are linked to intravenous drug use or the implantation of prosthetic valves [28]. At this point, one needs to mention pleuropulmonary infections as, next to *Pseudomonas aeruginosa, S. aureus* is one of the most serious causes of hospital-acquired pneumonia (HAP), including ventilator-associated pneumonia (VAP) and healthcare-associated pneumonia (HCAP) [29,30].

Infections involving the application of prosthetic devices and the use of catheters are mostly characterized by the presence of a biofilm. This structure can be defined as a microbial sessile community formed by cells attached to a particular surface (or to each other), surrounded by a specific matrix of an extracellular polymeric substances (EPS) [31].

In comparison to planktonic bacteria, biofilm-producing bacteria have a different phenotype and their gene expression, as well as protein synthesis, are remarkably different. Furthermore, the thickness of a biofilm can range from a very thin, single cell layer to a massive, multidimensional structure covered with a viscous polymeric milieu [31]. It should be emphasized that EPS can be composed of water, polysaccharides, microbial cells, and other extracellular products that may affect its structural integrity and stability [32]. Also, extracellular DNA (eDNA) appears to be one of the crucial biofilm components that plays a pivotal role in biofilm adhesion, gene transfer, and further survival [33]. For instance, as an anionic molecule, it can bind antimicrobial peptides (AMPs) produced by the immune system or other cationic antibiotics, such as aminoglycosides [34,35]. For *S. aureus*, the eDNA has been found to be one of the major structural components of the biofilm and it is likely to play a key role in its formation and virulence in vivo [36,37].

Interestingly enough, the presence of eDNA is not accidental. Some studies indicate that it can be secreted by metabolically active cells or can be obtained using controlled autolysis [38,39,40]. *S. aureus* shows a unique ability to form a biofilm on the surface of prosthetic devices. When such devices are implanted within the endovascular system, they are covered by the host proteins, such as fibrin, fibronectin, fibrinogen, and collagen [41,42,43,44,45]. Furthermore, *S. aureus* adheres to their surface initially via electrostatic interactions, van der Waals forces, and hydrogen bonds with subsequently more stable binding owed to the bacterial proteins of the MSCRAMM family (microbial surface components recognizing adhesive matrix molecules).

A great example of MSCRAMM is a fibronectin-binding protein A (FnBPA) that allows *S. aureus* to bind to a fibronectin-covered surface of external devices and also promotes adhesion to mucosal cells and tissues [46,47]. The first group of infections related to biofilm formation on prostheses are cardiac-device infections (CDIs) that are commonly associated with the implantation of permanent pacemakers (PPMs) or implantable cardioverter defibrillators [48]. Intravascular catheter infections, which are a common cause of sepsis and prolonged intravascular antimicrobial therapy, are another important group of infections characteristic of staphylococcal invasion [49,50]. However, the importance of orthopedic implant infections cannot be underestimated as the number of orthopedic surgical procedures, such as arthroplasties, has increased remarkably over the past decade.

Several studies indicate that *S. aureus* is the major pathogen involved in orthopedic infections [51,52,53,54]. Apart from infections strictly related to therapeutic processes, staphylococcal ones may also be associated with breast implantation. However, although only 1 to 2.5% of breast prostheses result in infection, *S. aureus* was found to be the dominant pathogen [55,56]. Although the knowledge about the resistance and virulence of *S. aureus* is extensive, the therapy against staphylococcal infections has been increasingly challenging. Another issue that needs to be resolved is the treatment of biofilm-associated infections. In view of their multiplicity and several etiological factors involved, different methods have been used to provide reliable data and to arrive at a successful application of innovative approaches in clinical management. This review looks at different methods applied to eradicate biofilms and to inhibit their formation. Moreover, since *S. aureus* (and MRSA) is a common pathogen associated with community and hospital-acquired infections, special attention has been paid to the approaches applied for staphylococcal biofilm elimination.

## 2. Laboratory Approaches for the Determination of Antistaphylococcal and Anti-Biofilm Activity

### 2.1. Standard Methods Used for the Determination of Antimicrobial Activity

**Figure 1:**
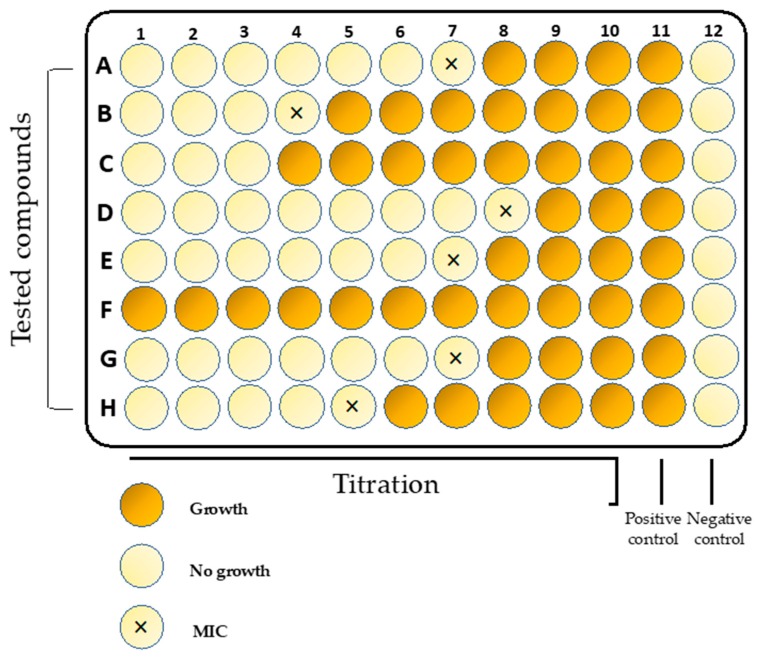
Example of microtiter plate and interpretation of microdilution results. MIC—minimum inhibitory concentration.

Newly developed compounds need to be tested using reproducible and reliable assays to discover their desirable properties. A broth microdilution method (Figure 1) is usually applied in in vitro susceptibility testing for *S. aureus*. For instance, the protocol issued by the Clinical and Laboratory Standards Institute (CLSI) is commonly used in scientific studies. First of all, it is standardized, recommends the use of a specific microbiological medium (Mueller–Hinton Broth), as well as the method for cultivating microorganisms and the size of the initial inoculum [57,58,59,60,61]. Second, it is internationally accepted by organizations, such as the European Committee on Antimicrobial Susceptibility Testing (EUCAST), the British Society for Antimicrobial Chemotherapy, the Deutsches Institut für Normung, and the Comité de l’Antibiogramme de la Société Française de Microbiologie.

The results obtained using this method are quantitative in terms of minimal inhibitory concentrations (MICs) expressed as the concentration of a tested compound at which no visible growth of bacteria is observed. The method is reproducible and allows for the use of panels and automated plate readers. However, it has some limitations [62]. For instance, it cannot be applied for hydrophobic molecules or for those that bind to the surface of polystyrene. For this reason, several modifications such as the use of polypropylene microtiter plates or glass tubes were introduced in order to overcome those limitations [63]. Another standardized approach also issued by CLSI is the disk diffusion method. The test is performed by applying a bacterial inoculum to the surface of a large (in most cases 150 mm in diameter) Mueller–Hinton agar plate. Subsequently, a few paper disks fixed with a specific concentration of the tested compounds are placed on the inoculated agar surface and incubated. The diameter of the growth inhibition zone around each disk is related to the susceptibility of the isolate and to the diffusion rate of the drug through the agar medium. This method is routinely applied in laboratory medicine to determine the susceptibility profile of clinical isolates to conventional antibiotics. Moreover, it is also successfully used for the screening and investigation of plant extracts, quantum dots, and nanoparticles [64,65,66,67,68,69,70].

### 2.2. Minimal Biofilm Eradication/Eliminating Concentration Assays

**Figure 2:**
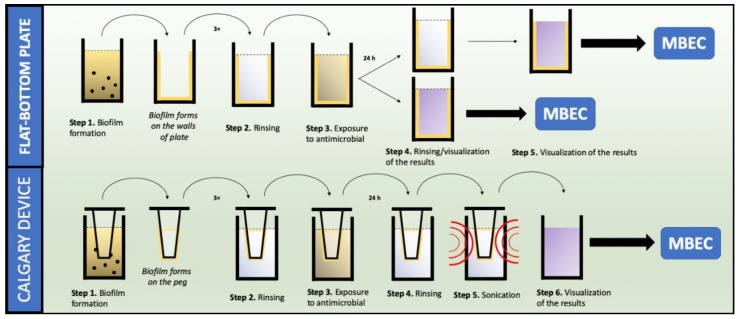
Schematic diagram of MBEC determination.

Biofilm formation plays a pivotal role in the development (up to 85%) and persistence of infections caused by *S. aureus* [71,72]. As the overall susceptibility of this structure is significantly higher compared to that of planktonic forms, it is important to consider its investigation while designing and examining new antimicrobial agents. Therefore, several in vitro approaches have been established to determine the susceptibility, as well as to characterize the *S. aureus* biofilm [73]. In fact, the majority of these assays utilize the high-throughput quantification of the biofilm with the use of microtiter plates and specific reagents. In comparison to MIC determination, these assays are often conducted using flat bottom or modified 96-well plates, called the Calgary device or high-throughput plates (the schematic diagram of both methods is presented in Figure 2) [74,75]. Both assays enable the determination of the minimum biofilm eradication concentration (MBEC) [76,77].

The ATP bioluminescence (BLM) assay is a method that takes advantage of the measurement of the intracellular concentration of ATP, which correlates with the number of viable bacteria cells and is related to different growth conditions [78]. It employs the bioluminescent reaction of ATP with firefly luciferase and also enables the detection of live bacterial biomass, as well as those bacteria with a low metabolic activity [79].

For staphylococci, a BLM assay was successfully applied in the quick bacteriophage-mediated detection of bacteria in a sonicated fluid of explanted artificial joints, in the rapid quantification of bacterial biofilm on vascular graft materials, and in the determination of antibiotics susceptibility of biofilms formed on microtiter plates [80,81,82]. Crystal violet (CV) and safranin (SAF) assays are commonly used for biomass quantification in biofilm-based research of *Staphylococcus* spp. [83,84,85]. Both methods are based on the use of specific dyes that penetrate through the biomass of pre-grown biofilms. After a specific time of incubation, the non-bound CV and SAF are removed, the particular solvents (30% solution of acetic acid or a mixture of acetone and ethanol and 0.1 M HCl, respectively) are added, and their release from biofilms is followed using absorbance measurements. As a matter of fact, neither method offers an answer to the question about the exact number of bacteria involved in biofilm production or about the number of killed bacteria, but they can provide information about the existence and size of the biomass (EPS) produced by individual strains. This feature is highly appreciated as it can be fundamental for the determination of the antimicrobial susceptibility of the biofilms [86].

**Figure 3:**
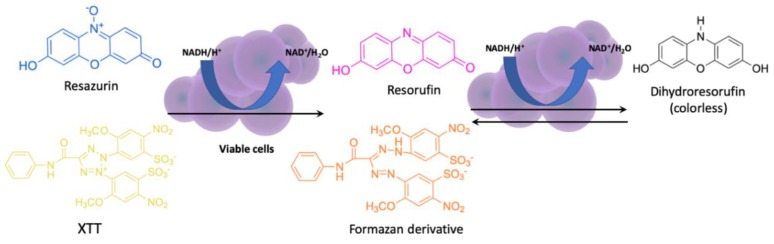
The principle of XTT and resazurin assay. NAD—nicotinamide adenine dinucleotide.

Although both methods have been interchangeably used in biofilm studies, they are not free from drawbacks. For example, CV is unstable during storage and is characterized by a high toxicity while SAF does not give a satisfying optical response [87,88]. Resazurin (RES) and sodium 3,3′-[1(phenylamino)carbonyl]-3,4-tetrazolium]-3is(4-methoxy-6-nitro) benzene sulfonic acid hydrate (XTT) assays are based on the metabolic reduction of the initial substrates, which is followed by a change of color. XTT is a kind of colorless or slightly yellow tetrazolium salt that can be reduced to a water-soluble, brightly orange formazan derivative. RES is a blue compound that is irreversibly metabolized to a pink fluorescent resorufin (Figure 3). The results of both assays allow for direct reading of the absorbance measurement, which makes both procedures efficient and intuitive. However, it should be emphasized that in the case of the RES assay, a prolonged incubation leads to a further reduction of resorufin to non-fluorescent dihydroresorufin [89], especially for fast metabolizing cells like *S. aureus*.

Interestingly, not only resazurin itself, but also several resazurin-based compounds, were used in biofilm examination. One of them, the Presto Blue cell viability reagent (Thermo Fisher Scientific, Waltham, MA, USA), was used in the screening of commercial pharmacologically active small compounds against *S. aureus* biofilms [90]. However, although RES and XTT both rely on metabolic activity measurements, they were found to not correlate with one another. Interestingly, in the research conducted by Alonzo et al. on 209 strains of *S. aureus*, a significant disagreement (61.2% with r = 0.024) between both methods has been reported [91]. Moreover, the correlation decreased when metabolic activity of the bacteria was taken into account, thus suggesting that only one approach should have been chosen for experiments on *S. aureus* biofilms. In fact, metabolic activity measurements and biomass determination have been found to be more appropriate and are frequently applied in parallel, often supplemented with microscopic imaging [91].

Nevertheless, to provide reliable and accurate data, sometimes the analysis of the differences between contrasting methods should be provided. For instance, in the report by Xu et al., the CV and XTT assays were compared to find differences in metabolic activity and biofilm production of a large number of clinical strains of *S. aureus* [90]. As a result, distinct strain-to-strain dissimilarities were found and the impact of several factors on biofilm formation was revealed. Consequently, the authors concluded that the combinatory approach in biofilm-related research is the best way to provide relevant data. Interestingly, not only colorimetric or fluorometric measurements can be applied in the assessment of biofilm eradication. Some studies indicate that piezoelectric sensors have also been found to be a convenient tool for biofilm development monitoring [92,93,94]. In this approach, the electrical resonance frequency increases due to the biofilm formation on a specific sensor. On the other hand, piezoelectric elements were also applied in prophylaxis [94]. For instance, Hazan et al. have demonstrated that piezoelectric elements attached to the outer surface of catheters generate low acoustic waves that cause vibrations throughout the medical device and the adjacent aqueous environment [95]. This resulted in a significant inhibition of biofilm formation. Visual imaging complements biofilm studies and allows one to follow how the structure is being formed and how exactly the eradication occurs.

Moreover, if specific dyes are used, it is easier to evaluate how a particular compound interacts with the biofilm. LIVE/DEAD staining is a method routinely used for antistaphylococcal activity visualization. Ready-for-use kits available in the market are more specific and well-validated for Gram-positive bacteria [96]. In this method, the staining mixture is composed of two fluorophores, namely SYTO9 and propidium iodide (PI). PI is a red-fluorescent DNA-specific stain that penetrates only cells with disrupted membranes. That is why it is used for the identification of dead cells [97]. SYTO9 is a green-fluorescent stain that enters both live and dead cells. However, its affinity to nucleic acids is lower than that of PI. Consequently, for dead cells it is replaced with PI and its fluorescence is reduced [98,99].

Moreover, the reduction of the SYTO9 signal is also connected with a fluorescence resonance energy transfer (FRET) [100]. For microscopic visualization, LIVE/DEAD staining is often used in conjunction with confocal laser scanning microscopy (CLSM), which allows one to visualize the whole biofilm spatially and more accurately. Furthermore, it can be applied in several approaches to provide information about biofilm behavior under different conditions. For example, Traba et al. used LIVE/DEAD staining for susceptibility monitoring of a *S. aureus* biofilm to reactive discharge gases [101,102]. Haney et al. used this method in their study to investigate the impact of media composition on staining patterns and the activity of antibiotics and antimicrobial peptides (AMPs) against *Pseudomonas aeruginosa* and *S. aureus* strains [103]. The CLSM imaging revealed that the MgSO_4_ composition can affect the biofilm architecture of *P. aeruginosa*.

On the other hand, it should be emphasized that both fluorophores can be used separately, depending on the purpose of their application. For instance, in the study by Sonesson et al. on staphylococcal enzymes (staphopains) and their proteolytic activity, the SYTO9 with atetramethylrhodamine (TAMRA)-labelled LL-37 peptide were used to determine the peptide binding to *S. aureus* cells [103]. Furthermore, LIVE/DEAD staining allowed for the investigation into how particular compounds may interact with a *S. aureus* biofilm. In the study by Verderosa et al., the combination of profluorescent fluoroquinolone-nitroxide hybrids and the SYTO9/PI staining indicated that the activity of nitroxide-functionalized antibiotics is based on EPS penetration [104]. Scanning electron microscopy (SEM) complements all biofilm-based research and has been extensively used for its high resolution and magnification. However, the main drawback of SEM is the need for sample dehydration, which can affect the biofilm structure.

For this reason, several approaches (such as ionic liquid coatings) were introduced to overcome those limitations [105,106]. Furthermore, SEM imaging was found to be useful in research on novel antimicrobials as it can directly indicate how they affect the cell morphology [107,108]. On the other hand, SEM is also used to confirm the presence of a biofilm. In the study by Nishitani et al., SEM and bioluminescent imaging allowed them to investigate the mechanism of *S. aureus* proliferation and stasis during implant-associated osteomyelitis [108]. Furthermore, SEM also proved to be a useful tool in studies on *S. aureus* implant-associated infections [109,110,111]. In the research by Coraça-Hubér et al., Minimum Biofilm Eradication Concentration-High Throughput Plates (MBEC™-HTP) were used as a surface for biofilm formation [112]. The model of infection was found to be fast and reproducible. As a result, rifampicin and daptomycin were found to be suitable therapeutics for the management of *S. aureus* biofilm infections.

### 2.3. Flow Systems

Flow systems are often used to replicate in vivo conditions as they allow for the control of nutrient delivery, flow, and temperature. They are also suitable for microscopic imaging and on-line monitoring of growth. It should be noted that flow plays a fundamental role in biofilm formation as it promotes the growth of those bacteria that have been attached to the surface and rinses off the unbound planktonic forms. However, it is still unknown which model is most suitable for biofilm examination.

Some studies indicate that it is not the growth conditions themselves but rather the expression of phenol-soluble modulin (PSM) surfactant peptides that is the key structuring factor for *S. aureus* and may be pivotal for biofilm strength and thickness [113]. On the other hand, Kim et al. have found that fluid flow represses the internal signaling (quorum sensing) of *S. aureus*, which can be associated with the elution of signaling molecules [114,115]. Nevertheless, several methods based on continuous flow have been introduced [116,117].

The first and the simplest one is the modified Robbins device (MRD), which consists of a square-channel pipe with sampling ports where the examined coupons are mounted. It allows for the formation of various microbial biofilms on diverse substrates under controlled flow conditions. For *S. aureus*, it was used, for example, in the determination of the biofilm removal efficacy of novel disinfectants or in the examination of gentamycin-loaded bone cements [118,119]. Drip flow reactors (DFRs) are also frequently used for biofilm examination and they are designed for studying biofilms under low shear conditions. DFRs consist of four parallel chambers with vented lids. Each chamber contains a coupon where the biofilm is formed. The main characteristic of DFR is its ability to form multiple identical biofilms on removable discs that may be suitable for the testing of novel antimicrobial compounds [120]. For instance, Agostinho et al. used this method to measure the growth and to analyze chronic wound MRSA biofilms [121]. Also, The Center for Disease Control (CDC) biofilm reactors are used for flow studies. The CDC biofilm reactor consists of eight polypropylene coupon holders suspended on a ultra-high molecular weight (UHMW)-polyethylene ported lid. The coupons can be tested under different shear conditions as they are combined with a disk that is attached to a magnet. When the reactor is placed on the top of a magnetic stirrer, the rotational speed can be adjusted. The rotation of the disk creates a liquid surface shear across the coupons. For *S. aureus*, the CDC reactor was used, among others, in the development of biofilms on polyether ether ketone (PEEK) membranes for further inoculation in the animal model of orthopedic implant biofilm-related infections [122].

Microfluidic devices (MDs) are considered to be the most promising platform for biofilm studies. First of all, they provide a closed system where the biofilm can be exposed to various hydrodynamic environments and to different factors. Second, they are characterized by low reagent and media consumption and allow for microscopic imaging. MDs can be fabricated from a wide range of materials, including glass, thermoplastic materials, and flexible elastomer-like polydimethylsiloxane (PDMS). The latter is routinely used in biofilm studies as it allows for the customization and functionalization of its surface [123,124,125].

### 2.4. Functionalization of Prosthetic Devices and Biomaterials

The rapid development of medical devices used for implantation and the resulting more frequent application of biomaterials in various fields of broadly understood medicine has caused an increasing problem of biomaterial-related infections [126,127]. Biomaterials can be used at various stages of medical care, as well as in prevention, diagnosis, or therapy [126]. They often offer a chance for normal functioning, improvement of life quality, and provide pain relief [128]. Biomaterial implantation triggers the host’s defense mechanisms and stimulates inflammatory mediators, such as oxygen and lysosomal enzymes. In the human body, the implant is naturally coated with plasma components, including extracellular matrix (ECM) proteins [129,130,131]. Then, the so-called race for the surface starts, engaging ECM proteins, the host cells, and bacteria [129,132].

Biofilm formation is a multi-stage process that starts with the adhesion of bacteria to the artificial surface [129,133]. One of the reasons for the rapid contamination of implanted biomaterials is the markedly lower number of bacteria required to colonize such a biomaterial than is the case with living tissue [126,134]. This may be due to the lack of vascularization, which makes the implant more sensitive to bacteria than human tissues and organs [126].

*S. aureus* is a microorganism that perfectly adheres to extracellular matrix proteins and plasma components, which makes it an effective biofilm producer and a leading etiological factor of infections associated with the use of medical devices [72,135,136]. A medical device can be described as a tool, apparatus, device, instrument, or a piece of equipment used for prevention, treatment, mitigation, and rehabilitation regarding a medical condition. It allows one to obtain information about a patient’s disease and medical condition [126,137].

Four classes of medical devices can be recognized based on the level of control required to ensure their safety and efficiency. Class I has a low risk for the patient and does not require any license. For some of them, basic standards are required (e.g., surgical instruments, dentistry materials). With Class II, safety and efficacy data are demanded (e.g., contact lenses, ultrasound scanners, medical catheters). Class III is characterized by a high potential risk for the patient (e.g., orthopedic implants, such as bone cement, hip implants, hemodialysis machines, and surgical mesh). Class IV is a group of medical devices that represents the greatest risk and requires detailed supervision (e.g., cardiovascular implants, pacemakers) [126,138].

The physicochemical properties of several biomaterials significantly affect biofilm formation [139]. The risk of implant-related infections (IRIs) may be connected with the material’s shape and size, hydrophilicity, changes on the surface, composition, and biocompatibility [129,140,141]. It should be noted that the majority of prostheses and medical devices are made of metals, polymers, ceramic products, composites, and natural components [142]. The therapy of IRIs requires the administration of high doses of antibiotics and/or replacement of the implant during another expensive and risky surgery, which is not always effective due to an increasing resistance to antibiotics and a high risk of reinfection of the new implant [126].

That is why several research groups are focused on the development of solutions that will help prevent infections associated with the use of all types of medical devices. The surface of biomaterials can be modified via modulation of their chemical or physical properties, for example, by polishing or coating it with a thin layer of material with completely different properties. Among promising techniques that can inhibit the initial attachment of planktonic cells to the surface of the biomaterial is coating the implants with antibacterial and anti-biofilm agents. Among the proposed strategies is the application of natural and synthetic compounds [143]. Examples include a hydroxyapatite coating used on titanium alloys or covering the implant with an antibiotic or another compound with antimicrobial properties [144].

According to some authors, the delivery of antibiotics to the site of implantation results in enhanced effectiveness at a higher dose and allows for avoiding systemic toxicity. For instance, a promising antimicrobial efficacy against *S. aureus* was found for titanium implants coated with vancomycin and ceftriaxone [145]. Moreover, favorable properties of photoactive coating or coating with disinfectants were also noted, for example, for intramedullary implants and catheters [143,146]. Another approach is the use of enzymes.

For instance, Thellinger et al. observed a 70% reduction of a *S. aureus* biofilm created on a silicone urinary catheter coated with cellobiose dehydrogenase [147]. Other methods of catheter functionalization to prevent microbial access, both in vitro and in vivo, include coating with hydrophilic polymers, such as hyaluronic acid, hydrogels, heparin, or silanes [148,149].

Neut et al. have demonstrated a high degree of inhibition of *S. aureus* biofilm formation on gentamicin beads coated with biodegradable poly(trimethylene carbonate) [150]. Interestingly, polymer-lipid coatings containing antimicrobial peptides were also found to be safe and successful [151]. Some biomaterials may be treated with ceramic products, such as calcium phosphate with other biodegradable polymers [152]. Other strategies offering hope for limiting biofilm formation include biomaterial surface modifications using non-implantable devices.

The properties of the material itself can be changed without coating [126]. Clinical and laboratory indicators for diagnosing biofilm-related infections are determined by the patient’s medical history, symptoms of infection, the use of microscopic techniques, culture-based diagnostic techniques, and non-culture techniques, as well as the specific immune response against a given microorganism [153].

Therefore, traditional methods focus on the study of planktonic cultures. Microorganisms associated with a biofilm form aggregates that adhere to one another. Subsequently, the above results in chronic infections were followed by colonization of the tissues and surfaces of medical devices. For this reason, the bacteria may become more resistant to antibiotics and disinfectants and less capable of being detected in clinical specimens [154].

### 2.5. Methods for Detecting S. aureus Biofilms Formed on Biomaterials

According to European Society of Clinical Microbiology and Infectious Diseases (ESCMID) 2014 guidelines, the detection of a biofilm in samples requires a microscopic proof of infection, i.e., the presence of leukocytes, as well as confirmation that the microorganisms form aggregates in a matrix other than the surrounding tissue. Microscopic analysis can be carried out using light microscopy and routine staining methods, such as the Gram method, which allows for staining of tissues, mucous membranes, inflammatory cells, bacteria, and the biofilm matrix [153,155].

**Figure 4:**
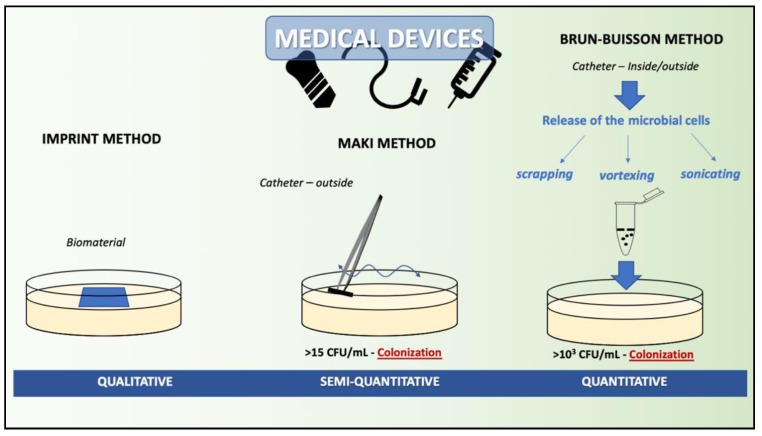
Methods of biofilm examination from medical devices.

CLSM and SEM are rarely used in routine microbiological diagnostics due to their limited availability. However, they provide interesting information about the microorganisms themselves and their interactions with a surface. Moreover, they provide data about the thickness of the biofilm, the surface of the biomaterial occupied by the biofilm, and finally about the bacterial metabolic activity. Interestingly, microscopic identification of samples can also be performed using the FISH (fluorescence in situ hybridization) method using a fluorescence microscope [153,156]. It should be noted that traditional culture and non-culture methods based on polymerase chain reactions (PCRs) do not distinguish between bacteria in the planktonic and biofilm forms [157,158]. The methods for examining a biofilm on biomaterials rely on the release of microbial cells from artificial surfaces (implants, medical devices). This can be done using the following methods [153,159,160,161,162,163]:

1. The imprint method where the biomaterial is imprinted on the surface of nutrient agar with visualization using Gram staining.
2. The roll-plate method, a semi-quantitative method according to D.G. Maki, that enables the detection of colonization of the external surface of a catheter/biomaterial. In this method, a sample of aseptically collected biomaterial should be rolled back and forth four times over the surface of agar with blood (plate with a diameter of 10 cm). After 24–48 h of incubation at 37 °C, the number of colonies on the surface of the plate is counted. Growth of more than 15 colonies of the microorganisms indicates colonization.
3. A quantitative method that allows for the calculation of CFU/mL, enabling the detection of external and internal colonization of a catheter/biomaterial. The most common is the Brun-Buisson method, which is performed in several ways: By vortex shaking the biomaterial in NaCl solution; by disruption using, for example, saponin; or sonication using ultrasound. After the recovery of microbial cells from the biofilm structure, the appropriate amount of the material is cultured on the nutrient medium, e.g., blood agar, on the biomaterial, and after incubation, the number of colonies per plate is calculated per 1 mL and the CFU/mL is obtained. The threshold value between colonization and contamination is 10^3^ CFU/mL (Figure 4).

The above-mentioned methods can be applied both in the diagnosis of biomaterial-related infections (BAIs) and in experimental procedures. For instance, sonication is one of the common methods used for biofilm removal. The time of sonication can vary from 1 to 30 min. Kobayashi et al. showed that a shorter time (1 min) was sufficient to remove *S. aureus* from a biomaterial. As a result, no differences in the CFU values for different times of sonication were noticed. Surprisingly, the combination of vortexing and sonication were characterized by larger amounts of detached biofilm [163].

## 3. Conclusions

The multiplicity of the methods that can be used for studying biofilms indicate how difficult it is to choose the most reliable one. In fact, not only the methods adopted for experiments, but also the growth conditions of the bacteria can be essential. *S. aureus* is responsible for a broad range of chronic and persistent infections that are still perceived as a serious threat to human health, especially given that they are associated with the presence of a biofilm [164,165].

As the treatment of *S. aureus* infections becomes more challenging, the research on novel antimicrobial strategies is all the more pressing. However, the choice of the appropriate method for a particular application can also be beneficial for further research. The present review summarizes the methods used most frequently in *S. aureus* studies. It should be emphasized that the basic research should rely on the initial examination of planktonic forms as the protocols are standardized. For biofilm studies, there is still a need for standardization [59]. However, not only the effectiveness of novel approaches should be taken into account, but also biofilm complexity and diversity. For this reason, the combinatory approach and the application of several methods is probably the optimal solution.
